# Mortality in Iranian Patients with Parkinson's Disease: Cumulative Impact of Cardiovascular Comorbidities as One Major Risk Factor

**DOI:** 10.1155/2015/834796

**Published:** 2015-10-20

**Authors:** Seyed-Mohammad Fereshtehnejad, Azadeh Shafieesabet, Mahdiyeh Shafieesabet, Gholam Ali Shahidi, Ahmad Delbari, Johan Lökk

**Affiliations:** ^1^Division of Clinical Geriatrics, Department of Neurobiology, Care Sciences, and Society (NVS), Karolinska Institutet, 14186 Stockholm, Sweden; ^2^Firoozgar Clinical Research Development Center (FCRDC), Firoozgar Hospital, Iran University of Medical Sciences, Tehran 15937-48711, Iran; ^3^Students Scientific Research Committee (SSRC), Faculty of Medicine, Tehran University of Medical Sciences, Tehran 14176-14411, Iran; ^4^Medical Student Research Committee (MSRC), Faculty of Medicine, Iran University of Medical Sciences, Tehran 14496-14535, Iran; ^5^Movement Disorders Clinic, Department of Neurology, Faculty of Medicine, Iran University of Medical Sciences, Tehran 14496-14535, Iran; ^6^Iranian Research Center on Aging, University of Social Welfare and Rehabilitation Sciences, Tehran 19857-13834, Iran; ^7^Department of Geriatric Medicine, Karolinska University Hospital, 14186 Stockholm, Sweden

## Abstract

Mortality rate, life expectancy, survival, and the impact of comorbidities on them in people with Parkinson's disease (PD) need to be assessed in settings with different sociodemographic backgrounds. We investigated mortality features in Iranian PD patients focusing on the role of cardiovascular multimorbidity on their survival. Data on mortality and comorbidity profile was gathered in a cohort of 190 individuals with idiopathic PD referred to a Movement Disorders Clinic. Standardized mortality ratio (SMR) compared to the Tehran general population was 3.44 and the life expectancy at birth was 67.4 (95% CI: 59.1–75.8) yr. Patients with at least one cardiovascular comorbidity had a shorter survival time after PD onset (14.0 versus 29.2 yr, *p* = 0.012). The hazard ratio for death increased 2.8 times (95% CI: 1.5–5.2, *p* = 0.002) with one additional cardiovascular comorbidity. Among all comorbid conditions, stroke showed the strongest independent effect on mortality in PD patients [HR = 13.1 (95% CI: 2.4–71.7), *p* = 0.003]. Conclusively, life expectancy was slightly lower in Iranian PD patients compared to the general population, while the SMR was high. Cardiometabolic multimorbidity substantially decreased survival in people with PD. Our study highlights the need for assessment, prevention, and treatment of cardiovascular morbidities in parkinsonian patients, given their effect on survival.

## 1. Introduction

Parkinson's disease (PD) is not a life-threatening illness by itself, but it may augment the risk of death through advanced complications such as aspirations, deep vein thrombosis, and pulmonary embolism during the end-stage phase in immobile patients or by serious falling in moderate-to-severe cases [1]. Even though the rate of progression considerably varies from one PD patient to another, they are expected to experience often about the same life expectancy as for the general population particularly among those who do not develop dementia and receive timely management and care [2]. Based on a recent meta-analysis, most studies reported increased mortality in PD (compared to general population) with a ratio ranging from 0.9 to 3.8 [3–6], while some others showed lower ratios, too [7, 8].

Several factors should be considered while studying mortality features in PD patients including methodological issues, study design, choice of study population, and comorbid conditions. End-stage PD patients are often frail elderly with high potentiality of having other chronic diseases; however, the role of comorbid disorders as a potential important driver for higher mortality has been commonly ignored in people with PD. It is still unclear how and to what extent does cardiometabolic and cerebrovascular multimorbidity affect life span and survival in PD patients. On the other hand, differences in life expectancy and mortality rates of general population from countries with diverse sociodemographic characteristics necessitate the evaluation of mortality features in PD patients who are selected from different populations. Therefore, we aimed at firstly investigating mortality features in Iranian patients with PD and then focusing on the role of cardiometabolic and cerebrovascular comorbidities on survival in PD.

## 2. Methods and Materials

### 2.1. Setting and Participants

This study was performed during January 2012 and September 2012 as a collaborative project between Karolinska Institutet, Stockholm, Sweden, and Iran University of Medical Sciences (IUMS), Tehran, Iran. Target study population was defined as Iranian patients with idiopathic Parkinson's disease (IPD) and study samples consisted of 190 IPD patients recruited from an outpatient referral Movement Disorder Clinic in Tehran, Iran. IPD was diagnosed by a neurologist specialized in movement disorders using the UK Brain Bank criteria [9]. In this project, all registered IPD patients during 2009-2010 were reassessed using their medical records and filling a checklist through telephone interviews.

### 2.2. Assessment

Patients' age at PD diagnosis, current age, or age at the time of death, sex, family history of parkinsonism, level of education, marital status, and treatment protocol were recorded. Moreover, information on comorbid conditions including heart failure, hypertension, diabetes, stroke, respiratory diseases, depression, and dementia was also gathered for each case. Patients' recent health status as dead or alive was determined through telephone interview. A new variable was created counting the total number of cardiovascular comorbidities consisting of heart failure, hypertension, diabetes, and stroke, which vary between 0–4.

### 2.3. Statistical Analysis

Data were analyzed using SPSS software version 17.0 (Chicago, IL, USA) and LifeTab Excel softwares. In order to describe continuous and categorical variables, mean [standard deviation (SD)] and frequency (percentage) were used, respectively. Based on the number of death events in each age category, age-specific mortality rates were calculated and then standardized using “*WHO World Standard Population*” as described elsewhere [10]. Following the extraction of life table, life expectancy at birth (*e*_0_) and its 95% confidence interval (CI) were calculated using the method recommended by “*Office for National Statistics*” (ONS) [11]. Kaplan-Meier analysis was applied to compute the mean survival time after the time of disease onset in IPD patients. We used log-rank statistic to compare the mean survival time between study subgroups. Multivariate Cox regression model was performed to indicate the factors that could independently affect survival time after PD diagnosis from a list of comorbid conditions and baseline characteristics entered into the model as predictors. In all analytical procedures, a two-sided *p* value < 0.05 was considered as the statistical significant level.

## 3. Results

### 3.1. Baseline Characteristics

Data from 190 patients consisting of 131 (68.9%) males and 59 (31.1%) females were assessed. The mean age at the onset of PD was 55.5 (SD = 12.1) yr and the average duration of disease was 8.1 (SD = 5.0) yr ranging between 1 and 30 years.  Table 1 summarizes the baseline and clinical characteristics of the entire study population. Majority of patients (96.3%) were under treatment with levodopa, while deep brain stimulation (DBS) was performed only in 8 (4.2%) cases. Depression (46.0%) and hypertension (20.1%) were the most common comorbidities.

### 3.2. Mortality Rate and Life Expectancy

**Figure 1:**
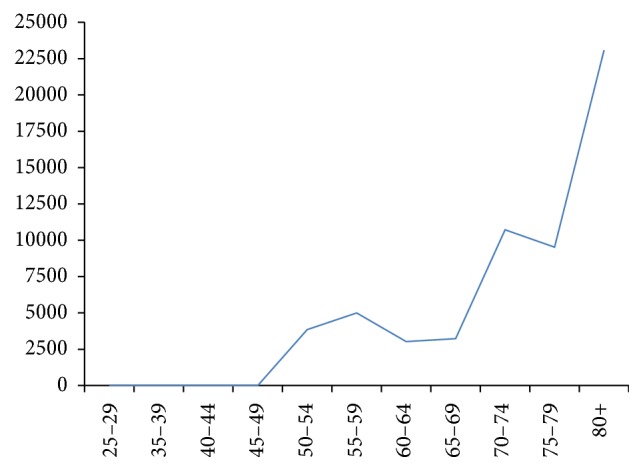
Age-specific mortality rates (per 100,000) in recruited Iranian Parkinson's disease patients.

Twelve patients were dead at the time of investigation and the mean age at the time of death was 71.4 (SD = 10.6) yr ranging from 51 to 87 years. As shown in Figure 1, the age-specific mortality rate raised gradually by age with a large increase in the “70–74 yr” age category. Following adjustment with “*WHO World Standard Population*,” the age-standardized mortality rate was calculated as 2777.9 per 100,000 (95% CI: 1206.2–4349.7/100,000) in the whole study population, 2481.3/100,000 for the males and 4885.1/100,000 for the females. Considering recent data on age-specific mortality rate in Tehran general population [12], the standardized mortality ratio was calculated as 3.44 in recruited PD population. Using the ONS method on life table data, the life expectancy at birth was computed as 67.4 (95% CI: 59.1–75.8) yr in the entire PD population, 70.2 (95% CI: 59.2–81.2) yr in male PD patients, and 64.9 (95% CI: 50.7–79.2) yr in female PD patients.

### 3.3. Survival Analysis

**Figure 2:**
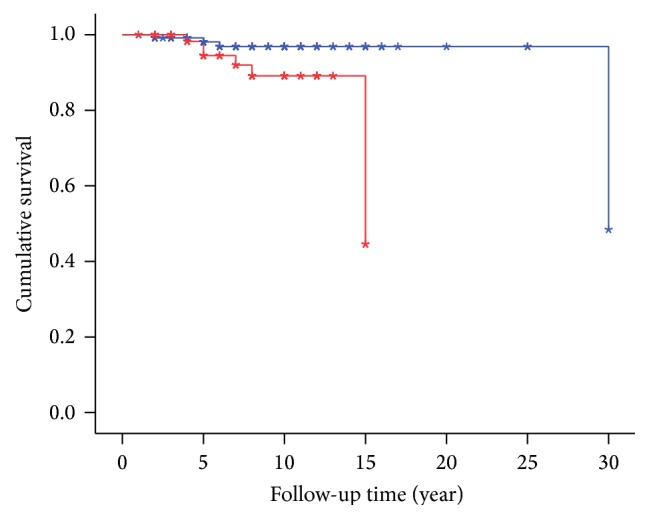
Survival curve in Parkinson's disease patients with (red line) and without (blue line) any cardiovascular comorbidity (*p* value for log-rank test = 0.012).

**Figure 3:**
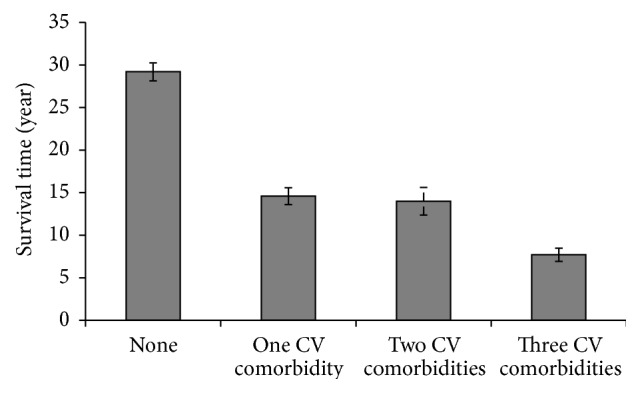
Mean survival time in recruited patients after Parkinson's disease diagnosis regarding the presence and/or number of cardiovascular comorbidities (*p* value for log-rank test < 0.001).

Kaplan-Meier method showed that the mean survival time after PD diagnosis was 25.7 (95% CI: 22.4–29.0) yr. Though the mean follow-up time was shorter, there were few patients with as long as 30 years of follow-up who had not met the outcome yet. These patients have been taken into account for the prediction modeling of the survival analysis.  Table 2 shows the findings from multivariate Cox regression models. Among all comorbid conditions, stroke had the strongest independent effect on mortality in PD patients [HR = 13.1 (95% CI: 2.4–71.7), *p* = 0.003]. History of having any cardiovascular comorbidity significantly increased the hazard for death [HR = 4.6 (95% CI: 1.1–18.7), *p* = 0.033]. As illustrated in Figure 2, PD patients with at least one cardiovascular comorbidity had a shorter survival time after the time of PD onset [14.0 (95% CI: 13.2–14.9) yr versus 29.2 (95% CI: 28.2–30.2) yr]. Result from the log-rank test demonstrated that this difference is statistically significant (Chi^2^ = 6.4, *p* = 0.012). Cox regression model also demonstrated that with one extra number of cardiovascular comorbidities the hazard for death significantly increased by 2.8 times (95% CI: 1.5–5.2, *p* = 0.002). As illustrated in Figure 3, the longest survival time after PD diagnosis was seen among those without any cardiovascular comorbidity [29.2 (95% CI: 28.2–30.2) yr], whereas the shortest survival time was calculated for those with three cardiovascular comorbid diseases [7.7 (95% CI: 6.9–8.4) yr]. These differences were also shown to be statistically significant by log-rank test (Chi^2^ = 18.2, *p* < 0.001).

## 4. Discussion

Our study showed slightly lower (≈1 yr) life expectancy at birth in Iranian PD patients compared to the reports for general Iranian population at almost the same time point (2011-2012), which has been estimated to be 68.6 and 71.6 yr in males and females [13], respectively. However, our sample size for a more valid calculation of life expectancy is rather small and its wide confidence interval includes the estimations for general population. Considering mortality rate in general population of Tehran, where the referral outpatient clinic is located, the SMR for recruited PD patients in our study was estimated as 3.44. Previous investigations have demonstrated a wide range of SMR for different PD populations ranging from 1.52 in Norway [14] to 3.38 in Taiwan [15] showing that our estimation for this group of Iranian PD patients is a high SMR.

We investigated the impact of cardiovascular comorbidities on survival in patients with PD, which demonstrated that the presence of cardiovascular comorbidity considerably reduces survival and increases hazard ratio for death by approximately five times. In a so-called “dose-response” effect, an addition of one cardiometabolic and/or cerebrovascular comorbidity accompanied with extra three times higher hazard risk of death in PD patients. Among all comorbid conditions, stroke showed the strongest independent adverse effect on mortality in people with PD. Furthermore, in this retrospective cohort of PD patients, we found that older age and female gender were associated with higher mortality risk. Though the independent effect of female sex on shorter survival time in our study was not statistically significant in further multivariate analysis, there are some few recent studies investigating gender differences in PD demonstrating a more severe course of PD in female patients [16]. More severe dyskinesia [17], more postural instability [18], more frequent freezing [19], more levodopa resistance [17], higher prevalence of depression [20, 21], and worse quality of life [22] could all contribute to shorter survival in female PD patients.

There are several studies that have investigated predictors of survival in PD, which resulted in inconsistent findings regarding the list of variables that they have measured. Yet, few of them have focused on the role of cardiovascular multimorbidity on mortality and survival in parkinsonian patients. One study has reported that terminal PD patients were hospitalized more frequently due to cardiovascular diseases and infection and rarely for PD-related complications [23]. Recent epidemiologic investigations have suggested that PD itself is associated with certain vascular risk factors such as diabetes [24, 25] and hypertension [26]. In our study, PD patients with at least one cardiovascular comorbidity had substantially shorter survival time after the time of PD onset (14 versus 29 yr). Similar to our findings, Ben-Shlomo and Marmot have reported that both ischemic heart disease (HR  =  2.3, 95% CI: 1.5–3.4) and stroke (HR  =  3.6, 95% CI: 2.2–6.1) significantly increased hazard ratio for mortality in a cohort of 220 PD patients [27]. On the contrary, another study showed no difference in the risk of death due to ischemic heart disease in PD patients after adjustment for age and gender (relative risk = 1.1, 95% CI: 0.6–2.0) [7].

In line with our findings from PD patients, researchers have quite recently demonstrated that any combination of cardiometabolic comorbidities such as diabetes, heart failure, and stroke associated with multiplicative mortality risk and reduced life expectancy in general population [28]. A cohort of 6504 individuals in Iran revealed that cardiovascular disease mortality constituted 31.8% of all-cause mortality in both men and women [29]. Some studies have enquired cardiovascular risk in individuals with and without PD. In a prospective population-based survey, cardiovascular diseases were the most frequent cause of death in persons with PD, and, in this respect, PD cases were similar to general population [30]. In another longitudinal study, cardiovascular diseases were found to be the first cause of death, which were attributed to the probable involvement of myocardium through autonomic dysfunction in PD [31, 32].

We acknowledge the limitations of our study of which the most important one refers to the limited list of variables that we have assessed. Several different combinations of risk factors for mortality in PD have been evaluated; however, we have mainly focused on the general features of mortality in Iranian PD patients (i.e., life expectancy and mortality rate) and the role of cardiovascular multimorbidity on their survival. Validity of information could have been also improved using more sophisticated methods such as full neuropsychiatric assessment for cognitive status. This might explain why, in contrast to previous studies that have introduced dementia as an important risk factor for shorter survival in PD [23, 33], our investigation failed to show such association, which is mainly due to lack of valid data on cognitive decline. Recruitment of patients from a specialist clinic where extreme frail elderly and those with lower socioeconomic status were less likely to be referred might have restricted generalizability of our findings. The proportion of participants who had not had the outcome during the follow-up (censored data in survival analysis) is high meaning that a longer follow-up duration is needed for more robust estimation of mortality features in PD patients. More in-depth information about the exact cause of death in PD patients obtained from national death register could be also helpful.

In conclusion, we showed that life expectancy might be slightly lower in Iranian PD population and their SMR is up to 3.44 compared to general population. Cardiometabolic multimorbidity played an important role in PD patients' survival such that each extra cardiovascular comorbidity substantially increased risk of mortality. Our study highlights the need for prevention and treatment of cardiovascular disorders in parkinsonian patients, given their effect on survival. Regarding the important role of cardiometabolic morbidities, PD patients should confer protection against the risk factors and consequences of common cardiovascular diseases. Although neurologist care associates with improved clinical outcomes and greater survival, less than half of PD patients are referred to them [34] and it could be a possible contributor to poor management of PD symptoms and less precise screening of other common illnesses. Moreover, physicians and patients may impute nonspecific symptoms such as fatigue, weakness, or exercise intolerance to PD, while they in fact represent other illnesses, like cardiovascular diseases.

## Figures and Tables

**Table 1:** Baseline and clinical characteristics of the recruited Parkinson's disease patients.

Characteristics	Total (*n* = 190)
Current age (or death age) (yr) mean (SD)	63.6 (11.0)
Age at the time of diagnosis (yr) mean (SD)	55.5 (12.1)
Sex *N* (%)	
Female	59 (31.1)
Male	131 (68.9)
Family history of parkinsonism *N* (%)	31 (16.4)
Level of education *N* (%)	
Illiterate	17 (9.1)
Primary and/or secondary	63 (33.7)
High school/diploma	51 (27.3)
College and/or university	56 (29.9)
Marital status *N* (%)	
Single	9 (5.6)
Married	127 (78.9)
Widow or divorced	25 (15.5)
Treatment history *N* (%)	
Levodopa	181 (96.3)
Amantadine	70 (37.2)
Selegiline	16 (8.5)
Bipyridine	6 (3.2)
Other dopaminergic drugs (i.e., ropinirole and pramipexole)	58 (30.9)
Deep brain stimulation (DBS)	8 (4.2)
Comorbidities *N* (%)	
Heart failure	34 (18.0)
Hypertension	38 (20.1)
Diabetes	24 (12.7)
Stroke	10 (5.3)
Respiratory diseases	20 (10.6)
Depression	87 (46.0)
Dementia	36 (19.0)

**Table 2:** Cox regression models to find independent factors predicting mortality in recruited Parkinson's disease patients.

Variables	Model 1				Model 2				Model 3			
	Hazard ratio (HR)	95% CI for HR		*p* value	Hazard ratio (HR)	95% CI for HR		*p* value	Hazard ratio (HR)	95% CI for HR		*p* value
		Lower	Upper			Lower	Upper			Lower	Upper	
Male gender	0.79	0.17	3.66	0.766	0.60	0.16	2.22	0.442	0.56	0.15	2.08	0.385
Dementia	0.62	0.06	6.19	0.685	0.45	0.05	3.89	0.470	0.51	0.06	4.58	0.549
Depression	0.51	0.12	2.09	0.346	0.63	0.17	2.32	0.491	0.49	0.12	1.93	0.306
Respiratory diseases	0.22	0.02	3.32	0.275	0.71	0.08	5.99	0.754	0.46	0.05	4.27	0.498
Hypertension	2.74	0.60	12.64	0.195	—	—	—	—	—	—	—	—
Diabetes	0.87	0.13	5.61	0.882	—	—	—	—	—	—	—	—
Heart failure	2.61	0.57	12.10	0.219	—	—	—	—	—	—	—	—
Stroke	**13.13**	**2.40**	**71.74**	**0.003**	—	—	—	—	—	—	—	—
Having cardiovascular comorbidity	—	—	—	—	**4.60**	**1.13**	**18.72**	**0.033**	—	—	—	—
Number of cardiovascular comorbidities	—	—	—	—	—	—	—	—	**2.75**	**1.45**	**5.21**	**0.002**
